# Insights Into Genetics and Pathophysiology of Arrhythmogenic Cardiomyopathy

**DOI:** 10.1007/s11897-021-00532-z

**Published:** 2021-09-03

**Authors:** Brenda Gerull, Andreas Brodehl

**Affiliations:** 1 Comprehensive Heart Failure Center (CHFC), Department of Medicine I, University Clinic Würzburg, Am Schwarzenberg 15, 97078 Würzburg, Germany; 2 Heart and Diabetes Center NRW, Erich and Hanna Klessmann Institute, University Hospital of the Ruhr-University Bochum, Georgstrasse 11, 32545 Bad Oeynhausen, Germany

**Keywords:** Arrhythmogenic cardiomyopathy, Junctions, Sudden cardiac death, Cardiovascular genetics, Desmosomes, Dilated cardiomyopathy

## Abstract

**Purpose of Review:**

Arrhythmogenic cardiomyopathy (ACM) is a genetic disease characterized by life-threatening ventricular arrhythmias and sudden cardiac death (SCD) in apparently healthy young adults. Mutations in genes encoding for cellular junctions can be found in about half of the patients. However, disease onset and severity, risk of arrhythmias, and outcome are highly variable and drug-targeted treatment is currently unavailable.

**Recent Findings:**

This review focuses on advances in clinical risk stratification, genetic etiology, and pathophysiological concepts. The desmosome is the central part of the disease, but other intercalated disc and associated structural proteins not only broaden the genetic spectrum but also provide novel molecular and cellular insights into the pathogenesis of ACM. Signaling pathways and the role of inflammation will be discussed and targets for novel therapeutic approaches outlined.

**Summary:**

Genetic discoveries and experimental-driven preclinical research contributed significantly to the understanding of ACM towards mutation- and pathway-specific personalized medicine.

## Introduction

Arrhythmogenic cardiomyopathy (ACM) defines a spectrum of mainly familial/genetic diseases, which includes not only the classical right ventricular dominant form — arrhythmogenic right ventricular cardiomyopathy (ARVC) — but also biventricular and left ventricular dominant forms such as arrhythmogenic left ventricular cardiomyopathy (ALVC). From the first description by Marcus et al. [1] as arrhythmogenic right ventricular “dysplasia” (ARVD) and the recognition as mainly inherited disease, we have learned a lot about the clinical presentation, which is dominated by the occurrence of life-threatening ventricular arrhythmias and sudden cardiac death (SCD) often in young individuals, sometimes athletes. Prediction of those events occurs still as a major challenge in the clinical management. With identification of genetic causes and subsequent cardiogenetic family screening, it became even more apparent that asymptomatic carriers require lifelong clinical observation and risk stratification. However, it also brought up that besides the primary genetic cause, other genetic and non-genetic modifiers and external factors play a role in the disease process, which are still barely understood. On the other hand, there is increasing understanding about the role of cardiac intercellular junctions, which are the central pathogenetic frameworks in ACM, and how structural alterations in composition and remodeling are drivers of the disease. In this review, we will focus on recent advances in clinical risk prediction, genetics, and pathophysiology of the disease and explain how understanding of the pathogenesis drives future therapeutic approaches.

## Clinical Features

### Clinical Presentation and Diagnosis

ACM is considered a rare disease with a prevalence of 1:2000 to 1:5000 [2]. Affected patients often present with palpitations, pre-syncope, syncope, or SCD in their 2nd–4th life decade mainly due to ventricular arrhythmias. Males are more often clinically affected than females (3:1) [3]. Key features are a mixture of structural and electrical abnormalities (arrhythmogenic cardiomyopathy). However, the clinical diagnosis remains difficult, requires a comprehensive clinical evaluation, and is based on the 2010 consensus task force criteria (TFC) [4]. A definitive diagnosis requires two major criteria or one major and two minor criteria or four minor criteria from different of the six categories (depolarization abnormalities, repolarization abnormalities, arrhythmia, imaging, histology, and family history/genetics). Current TFC still focus on the classical right ventricular form (ARVC) where primary left-sided forms are under-recognized in particular by the imaging criteria. Other limitations are due to the subjectivity of visual assessment of wall motion abnormalities, while newer methods such as strain imaging would be more accurate [5]. Due to those limitations, recently “the Padua criteria” have been proposed [6•]: here, ACM has been defined as “a genetic heart muscle disease involving the right ventricle, left ventricle, or both, characterized by fibro-fatty replacement predisposing to global and/or regional dysfunction, and ventricular arrhythmias independent of the ventricular dysfunction.” Additionally, some other changes to the original TFC have been proposed, e.g., cardiac magnetic resonance (CMR) imaging (wall motion abnormalities as a minor criterion) and CMR (late gadolinium enhancement (LGE) as a major criterion) apply ventricular tachycardia (VT) morphology criteria to the premature ventricular complex (PVC) criterion, etc. However, as suggested by the authors, the Padua criteria require further clinical validation before their final clinical implementation.

Importantly, differential diagnoses should be considered which include not only cardiac sarcoidosis, myocarditis, idiopathic right ventricular outflow tract tachycardia (RVOT), and Brugada syndrome, but also changes seen in an athlete’s heart or overlaps with dilated cardiomyopathy (DCM).

### Histology

An important hallmark of the disease is the histopathology, which in experimental models as well as in post-mortem or explanted myocardial tissue of patients shows dynamic changes and distinct patterns of fibrosis compared to other cardiomyopathies [7]. However, in the early stages of the disease, the changes are often discrete and non-diagnostic, in particular when based on endomyocardial biopsies (EMB), due to segmental distribution and the fact that the disease starts from the epicardium and extends later on to the endocardium. The end-stage or “overt” disease stage is characterized by the replacement of cardiomyocytes with fibro-fatty tissue. Sometimes cardiomyocyte hypertrophy is also present [8]. More lately, inflammatory cells — mainly T-lymphocytes but also macrophages — are found nearby areas of fibro-fatty replacement [7, 9].

**Fig. 1:**
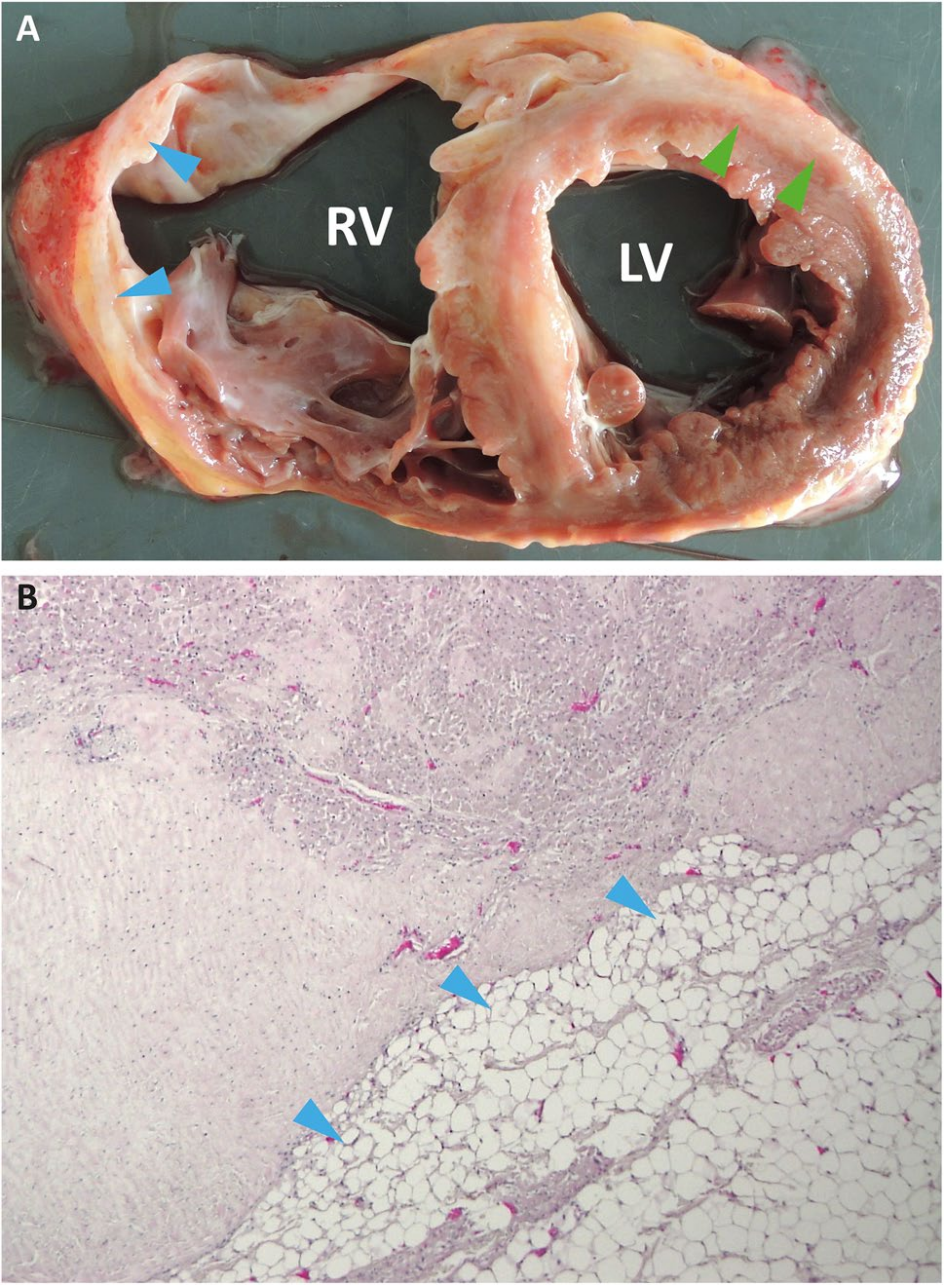
**A** Explanted heart of an ACM patient with biventricular cardiomyopathy and fibrous-fatty remodeling (arrows). Of note, the myocardial tissue of the right ventricle is nearly completely replaced against fibro-fatty tissue (blue arrows). However, fibro-fatty replacement is also present in the left ventricular myocardial tissue (green arrows). LV, left ventricle; RV, right ventricle. **B** Hematoxylin and eosin staining of myocardial tissue from an ACM patient. Fat replacement is marked with blue arrows

At the organ level, the “triangle of dysplasia” involving the right ventricular (RV) inflow and outflow tract and the RV apex is predominantly affected [4]. Left-sided disease is characterized by histological changes, which mainly affect the sub-epicardial layer or mid-mural layers of the free wall. Interestingly, even in clinically and genetically primary right-sided disease, post-mortem studies show also in half to two-thirds of the cases LV involvement at the histological level (Fig. 1A,B) [7, 10].

### Management

Curative treatment is not available to date. Management strategies focus on symptom relief, slowing disease progression and prevention of arrhythmias and SCD [11]. In addition, lifestyle interventions and in particular exercise restriction are recommended [12]. Exercise is an important risk factor. The mechanistic idea behind it is that intercellular junctions are exposed to mechanical stress and therefore further deteriorate their connections. According to the European Society of Cardiology, high-intensity sport should be discouraged because it accelerates disease progression and increases the risk for ventricular arrhythmias. A maximum of 150 min of low-intensity exercise per week should be considered for all individuals even in the absence of an overt disease phenotype [13].

Current medical therapy focuses on beta-blockers, which are recommended as first-line treatment to reduce the arrhythmic burden. However, if ineffective, class III antiarrhythmic drugs are recommended in selected cases, but none of them has shown to reduce the risk for SCD [14]. In late stages of ACM, standard heart failure treatment should be considered, and at end stage of the disease (heart failure or therapy-resistant arrhythmias) heart transplantation (HTx) [15]. In some patients with frequent monomorphic ventricular arrhythmias, radiofrequency catheter ablation therapy can be performed for symptom relief, but due to the progressive nature of the disease this may only be of short-term success.

### Risk Prediction

The average risk for patients with ACM to suffer from ventricular arrhythmias or SCD is 10% per year [16]. For the improvement of survival, the placement of an implantable cardioverter-defibrillator (ICD) is the only proven effective treatment. However, this invasive treatment with its own complications in often young active individuals requires careful considerations and a decision-making process of experts in the field. Expert statements and guidelines propose algorithms; the most recent one was published in 2019 from the Heart Rhythm Society where major and minor risk factors are defined and their presence or absence defines a strong, moderate, or weak indication for ICD implantation [11]. Lately, a new risk calculator (www.arvcrisk.com) has been introduced by a transatlantic initiative of experts aiming to improve ICD patient selection [17•]. The ARVC risk calculator predicts risk for fast (> 250 bpm) ventricular tachycardia/fibrillation or sudden cardiac arrest (VT/VF/SCA) based on four risk parameters (sex, age, T-wave inversions, PVC burden). In those individuals without a prior sustained event, seven risk parameters (sex, age, T-wave inversions, PVC burden, non-sustained VT, syncope, and right ventricular ejection fraction) can help predict the risk of any first sustained ventricular arrhythmias. Interestingly, prior sustained ventricular arrhythmias and the extent of functional heart disease are not associated with subsequent life-threatening ventricular arrhythmogenic events such as SCD.

## Genetic Causes and Modifiers

**Fig. 2:**
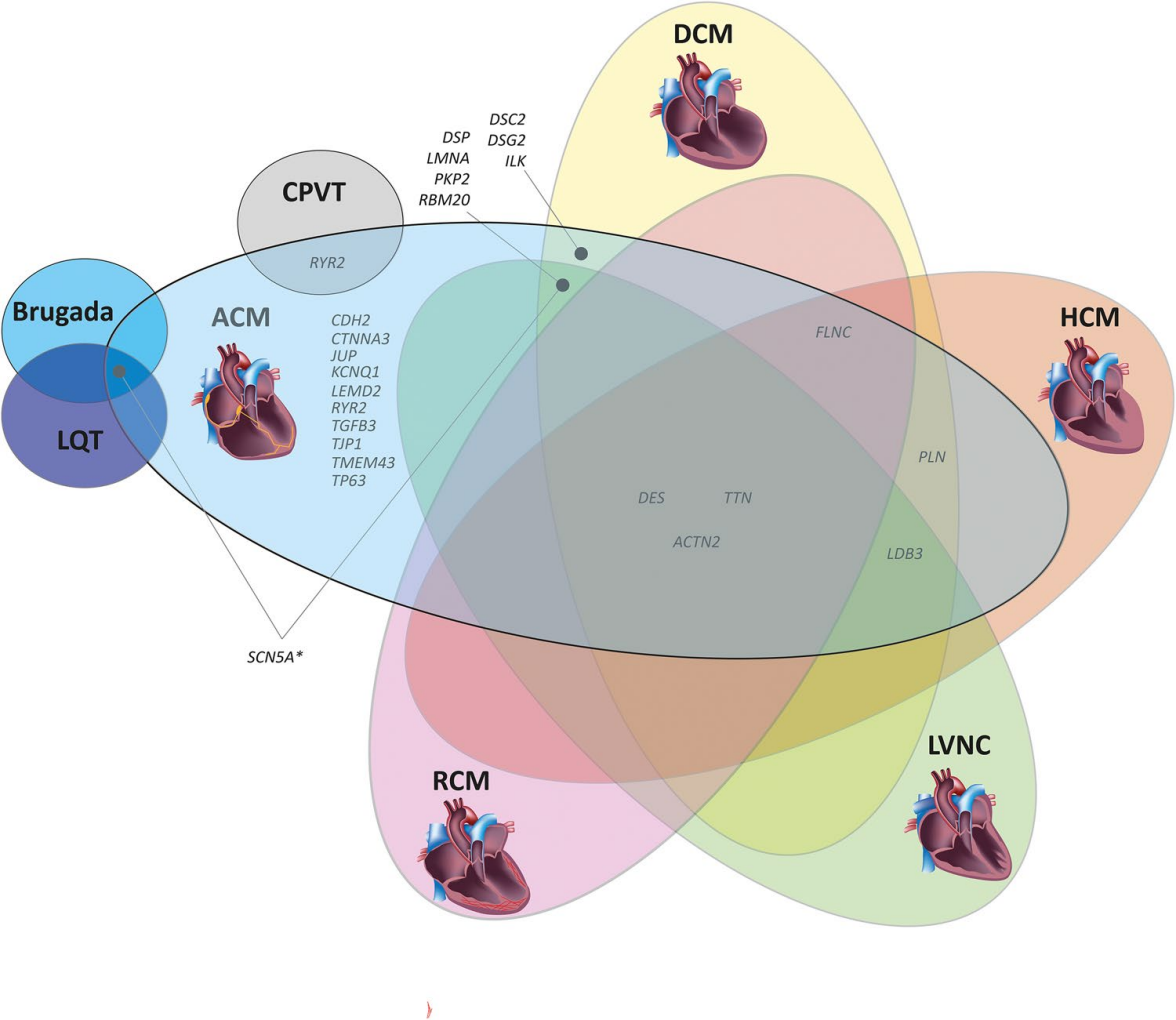
Genetic overlap of ACM with other cardiomyopathies and channelopathies. ACM, arrhythmogenic cardiomyopathy; DCM, dilated cardiomyopathy; HCM, hypertrophic cardiomyopathy; LVNC, left ventricular non-compaction cardiomyopathy; RCM, restrictive cardiomyopathy. Gene names according to HUGO Gene Nomenclature Committee, HGNC, https://www.genenames.org/. Sub-images of the DCM or HCM heart were licensed from shutterstock.com

**Fig. 3:**
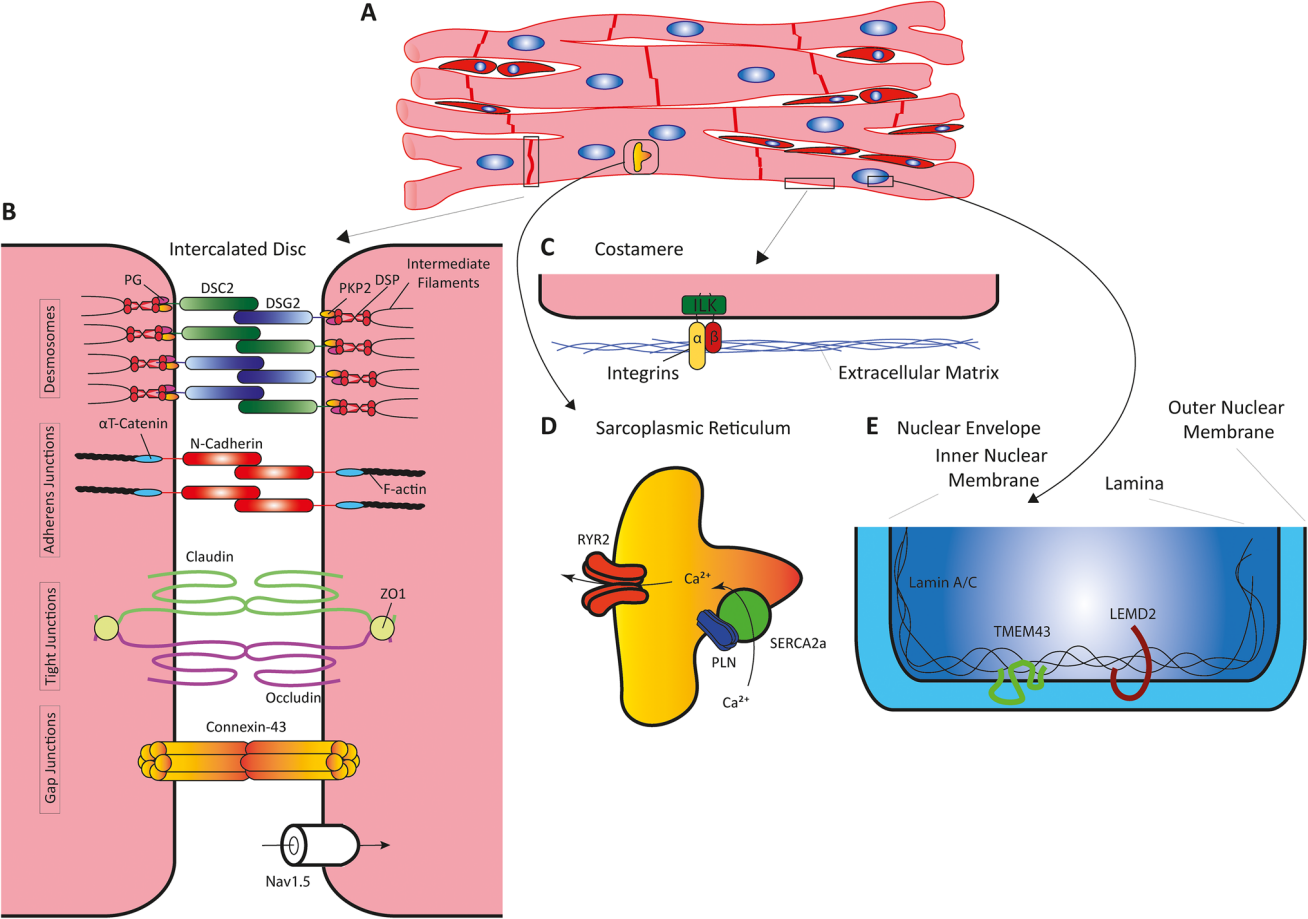
**A** Subcellular localization of proteins involved in ACM. Junctional multi-protein complexes involved in ACM, which are localized at the intercalated discs (**B**), at the costameres (**C**), sarcoplasmic reticulum (**D**), or the nuclear envelope (**E**). DSC2, desmocollin-2; DSG2, desmoglein-2; DSP, desmoplakin; PG, plakoglobin; PKP2, plakophilin-2

The majority of ACM patients have a family history of disease, indicating a genetic etiology. However, incomplete penetrance and variable expressivity ranging from mild phenotypes to severe cases including SCD are frequently observed even within the same family. In addition, de novo mutations and recessive, compound heterozygous, and digenetic inheritance can hide the genetic etiology in isolated index patients without familial history [18, 19]. Currently, mutations in more than 25 different genes have been described (for an overview, see Fig. 2) [20]. Since its genetic etiology is known and accounts for about 50% of cases, genetic diagnostics is highly recommended for patients and their relatives [11]. Most of the ACM-associated mutations are found in genes encoding proteins of different junctional multi-protein complexes like, e.g., the cardiac desmosomes localized in the intercalated disc (Fig. 3A,B).

### Junctional Gene Mutations

The generation of different knock-out, knock-in, and transgenic mouse models led scientists to discover that genes encoding for desmosomal proteins are highly relevant for the development of ACM [21–25]. Desmosomes are cell–cell junctions connecting cells exposed to nano-mechanical forces, such as cardiomyocytes during excitation–contraction coupling [26]. In the 2000s, it became evident that mutations in desmosomal genes cause ACM also in humans [27, 28]. The most common ACM-associated gene is *PKP2*, encoding plakophilin-2, which is a protein from the armadillo family [27]. These proteins have an armadillo domain, formed by different numbers of armadillo repeats each composed of three α-helices [29]. The majority of *PKP2* mutations are heterozygous nonsense, frameshift, or splice site mutations leading to haploinsufficiency. Plakoglobin, encoded by *JUP*, is a second armadillo protein involved in ACM [30]. However, mutations in *JUP* are rare and normally recessively inherited in a homozygous or compound heterozygous status. In addition to cardiomyopathy, *JUP* mutations also cause woolly hair and palmoplantar keratoderma, a triad of clinical symptoms known as Naxos disease (Mendelian Inheritance in Man, MIM, https://omim.org/, #601,214), since it was first recognized in families from the small Greek island Naxos [31]. Plakophilin-2 and plakoglobin bind to the cytoplasmic domains of desmoglein-2 (*DSG2*) and desmocollin-2 (*DSC2*), which are type I transmembrane proteins from the cadherin family. Hetero- and homo- or compound heterozygous pathogenic mutations in *DSG2* and *DSC2* have been described in about 5% of ACM patients [19, 32–34]. The majority of *DSG2* and *DSC2* mutations are localized in the extracellular domains, which consist of four cadherin and an anker domain, carrying several N-glycosylations and O-mannosylations [35, 36]. Hetero- and homophilic protein–protein interactions of the desmosomal cadherins are mediated by their first cadherin domains by strand swapping and are calcium-dependent [37]. On the intracellular side of the desmosomes, plakophilin-2 and plakoglobin bind to desmoplakin (encoded by *DSP*), which is a member of the plakin cytolinker family (Fig. 3B). Comparable to Naxos disease, mutations in *DSP* cause a combined cardiac and cutaneous syndrome, which is called Carvajal syndrome (MIM, #605,676) [38]. However, also cases with isolated ACM have been described [28]. Desmoplakin connects the cardiac desmosomes with the intermediate filaments, consisting of desmin in cardiomyocytes [39].

More recently, rare mutations in *CDH2*, encoding N-cadherin, were identified in ACM [40]. N-cadherin is localized at the intercalated discs but is part of the adherens junctions and of the *area composita*, which represent mixed-type junctions [41]. Adherens junctions are multi-protein complexes linked to the actin cytoskeleton and are similarly relevant for the structural integrity of the myocardium. Members of the catenin family bind to the intracellular domain of N-cadherin. Previously, rare pathogenic mutations in *CTNNA3*, encoding αT-catenin, were identified in ACM patients [42]. Li et al. reported that αT-catenin-deficient mice develop DCM in combination with ventricular arrhythmias [43].

Recently, the group of Rampazzo et al. identified mutations in *TJP1*, encoding the tight junction protein-1 also known as zonula occludens-1 (ZO-1), in a cohort of ACM patients [44]. Tight junctions are multi-protein complexes involved in sealing of the para-cellular space of adhering cardiomyocytes [45]. Different transmembrane proteins from the claudin and occluding family are the major building blocks of the tight junctions coupled by different scaffolding proteins with the cytoskeleton [46] (Fig. 3B).

The linkage of cardiomyocytes with the extracellular matrix (ECM) is mediated by costamere complexes, which are laterally localized in the sarcolemma (Fig. 3C). Integrins are important structural transmembrane proteins of the costameres and are connected by different adapter proteins with the cytoskeleton. Two pathogenic missense mutations in *ILK*, encoding the scaffolding adapter protein integrin linked kinase, have been recently described in patients with ACM [47]. In addition, more recently ACM-associated mutations in *FLNC*, encoding the large cytolinker protein filamin-C involved in coupling of the cytoskeleton with several cell–cell junctions, have been identified [48]. However, *FLNC* mutations cause also other types of cardiomyopathy [49].

### Non-junctional Gene Mutations

Besides mutations in genes encoding proteins of different cell junctional complexes, some other genes might be involved in ACM. However, there is less evidence of their pathogenicity and involvement in ACM than for junctional gene mutations.

#### Z-Band Proteins

*DES* encodes the cardiac intermediate filament (IF) protein desmin, and missense mutations in this gene cause right or biventricular forms of ACM [50–52]. However, the phenotypes associated with *DES* mutations are heterogeneous and range from isolated skeletal myopathies to different cardiomyopathies including DCM, left ventricular non-compaction (LVNC), and restrictive cardiomyopathy (RCM) [53]. Some patients present combined skeletal and cardiac muscle phenotypes [54]. IFs connect desmosomes and costameres, as well as Z-bands, mitochondria, and nuclei, and are therefore important for the structural integrity of cardiomyocytes. The primary consequence of pathogenic *DES* mutations is an abnormal cytoplasmic aggregation of proteins that alters the regular structure of the sarcomeres [55]. In addition, pathogenic mutations in *LDB3* and in *ACTN2*, encoding the Z-band proteins cypher and α-actinin-2, respectively, have been described in ACM patients [56, 57].

#### Proteins Involved in Cardiac Electrophysiology

Interestingly, mutations in genes encoding proteins involved in cardiac electrophysiology like *RYR2*, *SCN5A*, and *PLN* have been also identified in ACM patients [58–60]. *SCN5A* encodes the cardiac sodium voltage-gated channel subunit α5 (Na_v_1.5) and mutations in this gene are frequently found in patients with channelopathies like Brugada syndrome (MIM, #601,144) or long-QT syndrome (MIM, #603,830) [61, 62]. However, rare cases with DCM or ACM carrying *SCN5A* mutations have been described [63, 64]. The cardiac Na_v_1.5 channel is a large transmembrane protein consisting of four sub-domains, each formed by six transmembrane segments of SCN5A in combination with one β-unit [65, 66]. It mediates the inward sodium current that initiates the cardiac action potential [67]. *RYR2* encodes the ryanodine receptor-2, which forms a tetrameric calcium channel localized in the sarcoplasmic reticulum (SR) [68]. The ryanodine receptor 2 mediates calcium release from the SR and is thereby highly relevant for excitation–contraction coupling of cardiomyocytes (Fig. 3D). Besides ACM, mutations in *RYR2* have been linked with catecholaminergic polymorphic ventricular tachycardia (CPVT, MIM #604,772). The sarco-endoplasmic reticulum calcium ATPase 2 (SERCA2) pumps calcium back from the cytoplasm into the SR and is regulated in cardiomyocytes by phospholamban (encoded by *PLN*). Phospholamban is a small transmembrane protein localized in the SR membrane forming pentamers, which are structurally regulated by phosphorylation [69]. Recently, it was shown than *PLN* mutations cause both DCM and ACM [60].

#### Nuclear Envelope Proteins

Moreover, the nuclear envelope of cardiomyocytes is a sensitive cellular structure, which is currently affected by mutations in three genes/proteins associated with ACM (Fig. 3E). The nuclear lamina is a filamentous structure associated with the inner nuclear membrane and is involved in the three-dimensional organization and regulation of the genome [70]. Lamins are type V intermediate filament proteins and are the building blocks of the nuclear lamina [71]. Mutations in *LMNA*, encoding lamin A/C, cause different cardiomyopathies including ACM [72, 73]. At the molecular level, the ACM-associated mutation *LMNA*-p.L306R induces in vitro a hyper-assembly of recombinant lamin and induces nuclear structural defects [74]. Besides isolated cardiomyopathies, mutations in *LMNA* cause Hutchinson-Gilford progeria syndrome (MIM, #176,670), a systemic disease leading to premature aging including heart failure [75].

In Newfoundland, a predominant heterozygous founder mutation in *TMEM43-*p.S358L has been recognized as the genetic cause for an aggressive form of ACM mainly affecting males [76]. Although this *TMEM43* mutation has been found several times in other cohorts, the molecular function of this gene is unknown. Remarkably, the nonsense variant *TMEM43*-p.R372X causes auditory neuropathy spectrum disorder without causing ventricular arrhythmias or any other cardiac abnormalities [77]. *TMEM43* encodes the nuclear transmembrane envelope protein luma, which is a binding partner of emerin and lamins (see Fig. 3E). Several groups have generated different mice and zebrafish, and induced pluripotent stem cells carrying *TMEM43* mutations to model ACM [78•, 79–81]. However, both *Tmem43*-p.S358L knock-in and knock-out mice do not develop a cardiac phenotype under normal housing conditions [79] indicating that the pathogenicity of this specific mutation needs enhancement by overexpression or additional genetic, epigenetic, or environmental factors in mice.

Recently, the homozygous recessively inherited missense mutation *LEMD2*-p.L13R was found in patients from the Hutterite population affected by severe ACM in combination with cataract [82]. *LEMD2* encodes LEM domain containing protein-2, also known as nuclear envelope transmembrane protein-25. LEMD2 binds to lamin and is likewise involved in structural nuclear organization [83]. Comparable to *LMNA*, mutations in *LEMD2* cause also a progeria-related syndrome indicating pleiotropy for all three ACM-associated nuclear envelope encoding genes [84].

## Pathogenesis

Cardiac fibrosis and inflammation as well as cardiomyocyte death and hypertrophy are typical features of different types of cardiomyopathy. Although these pathophysiological processes are complex and contribute to several cardiac diseases, there is evidence that they have likewise high relevance for ACM.

### Cardiac Fibrosis

Fibro-fatty replacement of the myocardium, especially in the right ventricle, is a hallmark of ACM (Fig. 1) [85, 86]. In general, the development of cardiac fibrosis is a reparative process in response to injury by different triggers. However, its progression can cause patchy scar formation [87] and thereby increase stiffness and impair contractility of the myocardium. In ACM patients, cardiac fibrosis can be determined by CMR imaging using LGE [88, 89] or by EMB with classical histology [90]. In addition, it can be investigated ex vivo after HTx or autopsy [91, 92]. Different potential pro-fibrotic triggers have been identified in ACM. The fragility of cardiac desmosomes induced by genetic defects in combination with mechanical stress during exercise might contribute to cardiomyocyte injury as well as to pro-fibrotic and pro-inflammatory activation [93]. An abnormal structure of cardiac desmosomes and other multi-protein complexes of the intercalated disc have been described in explanted myocardial tissue from ACM patients, in animal models, and in cardiomyocytes derived from induced pluripotent stem cells (hiPSC) [34, 87, 94–96]. Because a complex interaction of different cardiac cell types like cardiomyocytes, myofibroblasts, and immune cells is involved, it is difficult to determine the cascades of molecular and cellular events leading to cardiac fibrosis in ACM. Recently, Maione et al. showed that the pro-fibrotic transforming growth factor β1 (TGFβ1) is overexpressed in ACM patients [97]. In good agreement, Dubash et al. showed that loss of plakophilin-2 increases TGFβ1 signaling [98]. Zheng et al. observed that the nuclear factor κB (NFκB) is hyper-activated in a *Tmem43*-p.S358L mouse model, leading to an increased TGFβ1 expression and consequently to cardiac fibrosis [99]. In this context, it is interesting that a rare mutation in the regulating promoter region of the *TGFβ3* gene has been described in ACM patients [100].

In addition, it has been suggested that signaling via the Wnt pathway leads to pro-fibrotic, pro-adipogenic, and pro-apoptotic gene expression changes in ACM [101]. β-catenin is a member of the Armadillo protein family and is an important signaling molecule within the Wnt pathway. It not only binds to the adherens junctions but can also shuttle into the nuclei, where it modulates transcriptional gene expression leading to pro-survival stimuli of the cardiomyocytes [102]. Cytoplasmic β-catenin is phosphorylated and degraded by the destruction complex containing the glycogen synthase kinase 3β (GSK3β). GSK3β is a serine/threonine kinase and a central mediator of the Wnt pathway. However, the contribution of the Wnt/β-catenin pathway in ACM is currently being debated [103]. An abnormal nuclear localization of plakoglobin, which is also known as γ-catenin, in combination with a decreased Wnt/β-catenin signaling has been associated with ACM in several studies [104–106]. However, other studies were unable to confirm the finding of an abnormal nuclear plakoglobin localization using myocardial tissue from ACM patients or ACM mouse models [107]. Nevertheless, inhibition of GSK3β by SB216763 causes a re-localization of plakoglobin, leading to increased survival of an ACM zebrafish model [108]. Some other studies using different ACM cell culture and/or animal models support this therapeutic effect of GSK3β inhibition [78•, 109].

Using human explanted heart tissue from four ACM patients and two different ACM mouse models as well as knock-down experiments of *Pkp2* in HL-1 cardiomyocytes, Chen et al. demonstrated a pathogenic activation of the Hippo pathway leading to increased adipogenesis [110]. The Hippo pathway consists of a cascade of different kinases activating YAP and TAZ, which are transcriptional coactivators of transcription factors from the TEAD family [111]. But, Rouhi et al. analyzed human tissue from ACM patients, carrying *DSP* truncation mutations, using RNA sequencing and revealed a suppression of the Hippo and Wnt pathway mediated gene expression at the mRNA level [112]. Also, Shoykhet et al. showed that activation of protein kinase C (PKC) and inhibition of the p38-MAPKinase pathways can increase cell–cell adhesion of the cardiomyocytes [113]. In summary, the pathological modulation of signaling pathways involved in ACM is complex. Different signaling pathways have an effect on each other and contribute to alterations in transcriptional and translational gene or protein expression patterns associated with ACM. To what extent the modulation of those signaling pathways will provide effective therapeutic strategies without side effects requires further evaluation.

### Loss of Cardiomyocytes by Apoptosis and Necrosis

Cardiomyocyte loss is a hallmark of ACM. Different kinds of cell death, like apoptosis, necrosis, or atrophy, have been suggested to explain loss of cardiomyocytes in ACM [7, 114–118]. Apoptosis is a programmed form of cell death induced by different triggers and is mediated by signal transmission pathways leading to the activation of cysteine-dependent aspartate-directed proteases (Caspases) [119]. Members of the Caspase family mediate a suicide cell death program without affecting neighboring cells [120]. Cell shrinkage and DNA fragmentation are the typical cellular and morphological features frequently observed in apoptosis [121], which can be determined in paraffin slices by TUNEL assays. During the 1990s, it became evident from studies using myocardial sections that apoptosis contributes to ACM [116, 122].

### Inflammation

Early studies of cardiac tissue from ACM patients detecting immune cell infiltrates and the release of pro-inflammatory cytokines suggested that chronic inflammation might play a role in the disease process [7, 9]. However, it remains unclear whether and to what extent these cell infiltrates represent tissue-resident immune cells. It is still debated whether myocardial inflammation promotes fibrosis and fibro-fatty replacement or if it is just a bystander and secondary to cardiomyocyte death.

More recently, clinical reports of genetically proven ACM patients presenting with a clinical picture of acute myocarditis are suggesting that the disease may pass different phases [123]. The so-called hot phases are characterized by chest pain, ST-elevation, and troponin release [124]. It seems that in particular genetic variants in the *DSP* gene are involved and that these acute events may require a trigger such as physical exercise [125•, 126].

Additionally, autoantibodies against desmoglein-2 were detected in ACM patients, independent of their genetic cause, and a positive correlation between antibody titer and disease severity and risk of arrhythmias was described [127]. A recent study also suggested the presence of anti-heart autoantibodies (AHAs) as well as anti-intercalated disk autoantibodies (AIDAs) in the majority of familial and in almost half of sporadic ACM cases and associated them in probands and clinically affected relatives with features of disease severity [128•]. Further independent and larger cohort studies are required to confirm these findings and assess the value of autoantibodies as a biomarker to predict disease severity and outcome.

The role of inflammation has been also addressed in murine models of ACM, where immune cell infiltrates, inflammatory cytokines, and chemokines and the activation of the pro-inflammatory nuclear factor κB (NF-κB) signaling pathway have been suggested to play a role in disease progression [129, 130]. The NF-κB inhibitor Bay 11–7082 mitigates the inflammatory response and ACM features in a Dsg2^mut/mut^ mouse model [131•]. This suggests that anti-inflammatory agents may also have a beneficial effect in ACM patients. As drugs targeting TNFα or IL-1β are already on the market and have shown to be effective in other cardiac diseases [132], these approaches should be also considered for ACM treatment in the future.

### Arrhythmogenesis

Life-threatening ventricular arrhythmias and SCD are the clinical hallmarks of ACM. Therefore, many pre-clinical studies in vitro and in vivo focused on the underlying pro-arrhythmic mechanisms in early and late stages of the disease.

In early disease, the so-called concealed phase, fatal arrhythmias are mechanistically related to sodium channel (Na_v_1.5) and connexin-43 (Cx43) remodeling. Both proteins are part of the intercalated disc structure (Fig. 3A,B) and require intact desmosomes for their appropriate function. Studies in human cardiac tissue [96] and murine models lacking Dsg2 and Pkp2 demonstrated decreased Cx43 expression and aberrant localization affecting cell–cell coupling as well as a reduced sodium current leading to decreased conduction velocity [133]. Both may cause ventricular arrhythmias in the absence of structural abnormalities of the myocardium. In addition, alterations in calcium handling have been proposed to contribute to the arrhythmic burden via early and late after-depolarization events secondary to SR calcium overload [134, 135]. Interestingly, flecainide has been suggested to reduce arrhythmias in ACM patients. Flecainide inhibits both the sodium channel and the ryanodine receptor 2, thus inhibiting the spontaneous calcium release from the SR, potentially accounting for the beneficial effects of this drug [136].

In the advanced stage of the disease, electrical disturbances occur due to the isolation of cardiomyocytes via fibrotic remodeling and scar tissue in combination with the events described above such as the sodium channel remodeling. Consequently, heterogeneous electrical conduction of surviving cardiomyocytes and reduced excitability due to a decreased sodium current combined with fibrosis lead to the formation of re-entrant circuits — the source of fatal arrhythmias [133, 137].

Given those mechanistic approaches, arrhythmias in ACM show similarities to arrhythmias found in Brugada syndrome and CPVT.

## Novel Therapeutic Approaches

Current management approaches focus on symptom relief, prevention of SCD, and standard heart failure therapy. Novel therapeutic approaches are on the horizon and may help prevent progression of the disease while focusing on underlying genetic and molecular mechanisms and the pathophysiology of ACM.

### Gene Therapy

As ACM is mainly a genetic disease, putative gene therapy approaches have been suggested. Recently, Shiba et al. presented proof-of-concept experiments using adeno-associated viruses (AAVs) encoding for desmoglein-2 to rescue the phenotype of cardiomyocytes derived from iPSCs carrying a homozygous nonsense mutation in the *DSG2* gene [138•]. In addition, studies from the Olson’s lab have recently shown that genome-editing technologies like CRISPR-Cas9 can be applied for the treatment of Duchenne muscular dystrophy (MIM #310,200) [139–142]. Although currently not directly applied to the treatment of ACM, it is likely that novel genome editing tools like Cas9 [143], base pair editors [144], or RNA-editing tools based on Cas13 [145] might be promising tools for gene therapeutic approaches in the future.

### Targeting signaling and inflammation

Thanks to a high-throughput chemical screen performed in zebrafish, the compound SB216763 has been identified as a GSK3β inhibitor. The use in several pre-clinical models has shown that SB216763 ameliorates ACM features, and in particular the development of fibrosis [108, 146•, 147]. Because SB216763 activates the Wnt/β-catenin pathway, clinical applications of this compound are limited by its pro-oncogenic activity [147].

Another therapeutic approach is targeting inflammation. As described above, activation of pro-inflammatory pathways including the NF-κB signaling pathway may play a role in disease progression. Interestingly, a NF-κB inhibitor prevents disease features and inflammatory responses in mice [131•]. However, more data are required to prove beneficial effects in other model systems.

Over the last years, the contribution of inflammation and auto-immune responses became more evident in ACM. Therefore, clinically approved anti-inflammatory agents such as the p38MAPK inhibitor losmapimod, the anti-interleukin-1β antibody canakinumab, and TNFα inhibition with pentoxifylline may help attenuate the disease. But to date, none of these agents has been tested neither in pre-clinical models nor in patients with ACM. Interestingly, off-label use of conventional immunosuppressive therapy with prednisolone and azathioprine was beneficial in a child presenting with a clinical picture of autoimmune myocarditis with [148].

Another therapeutic approach has been suggested by the group of Schinner*,* who designed a linking bicyclic peptide (Dsg2-LP) to cross-link Dsg2 molecules and rescue arrhythmias in an ACM mouse model [149].

## Conclusion

ACM is recognized as a primary genetic cardiomyopathy and an important cause of SCD especially in young people. The clinical picture is heterogeneous and appropriate diagnosis is often difficult, in particular in early disease stages. However, the risk for fatal arrhythmias runs through all disease states. The introduction of a new risk calculator based on four criteria (namely sex, age, T-wave inversions, and PVC burden) may help clinicians in the decision-making process to identify high-risk patients for ICD implantation — so far, the only management option showing improved survival. However, recent insights into the genetic etiology and pathophysiology using experimental pre-clinical models identified novel signaling cascades and cellular mechanisms towards targeted therapeutic strategies. Furthermore, the view on ACM as a complex genetic disease influenced by environment, comorbidities, trigger factors such as competitive sport, and the immune system require further considerations to unravel underlying causes of incomplete penetrance and variable disease expressivity. Finally, yet importantly, novel gene therapeutic approaches are on the horizon to treat the disease at the molecular trigger where it originates.
